# Well-ordered polymer nano-fibers with self-cleaning property by disturbing crystallization process

**DOI:** 10.1186/1556-276X-9-352

**Published:** 2014-07-15

**Authors:** Qin Yang, Zhuangzhu Luo, Sheng Tan, Yimin Luo, Yunjiao Wang, Zhaozhu Zhang, Weimin Liu

**Affiliations:** 1 Chongqing Institute of Green and Intelligent Technology, Chinese Academy of Sciences, Chongqing 400714, People’s Republic of China; 2 State Key Laboratory of Solid Lubrication, Lanzhou Institute of Chemical Physics, Chinese Academy of Sciences, Lanzhou 730000, People’s Republic of China

**Keywords:** Polymer, Nano-fibers/spheres, Controllable, Superhydrophobicity, Disturbing crystallization

## Abstract

Bionic self-cleaning surfaces with well-ordered polymer nano-fibers are firstly fabricated by disturbing crystallization during one-step coating-curing process. Orderly thin (100 nm) and long (5–10 μm) polymer nano-fibers with a certain direction are fabricated by external macroscopic force (*F*_blow_) interference introduced by H_2_ gas flow, leading to superior superhydrophobicity with a water contact angle (WCA) of 170° and a water sliding angle (WSA) of 0-1°. In contrast, nano-wires and nano-bridges (1–8 μm in length/10-80 nm in width) are generated by “spinning/stretching” under internal microscopic force (*F*_T_) interference due to significant temperature difference in the non-uniform cooling medium. The findings provide a novel theoretical basis for controllable polymer “bionic lotus” surface and will further promote practical application in many engineering fields such as drag-reduction and anti-icing.

## Background

Bionic superhydrophobic (self-cleaning) surfaces with micrometer-nanometer-scale binary structure (MNBS) have aroused great interest of science and engineering fields [1-3], which can be attributed to their potential application prospects such as drag reduction on ship hulls [4], anti-biofouling in maritime industry [5], and anti-icing for power transmission [6]. Their superhydrophobicity (a water contact angle (WCA) larger than 150° and a water sliding angle (WSA) less than 10°) strongly depends on MNBS structure [7,8]. In the past few decades, many conventional attempts have been done to fabricate superhydrophobic surfaces with MNBS structure, such as creating a rough and well-ordered metallic or inorganic surface covered with low surface energy molecules, which is called two-step methods [9-14]. However, these methods usually are applied to small-scale substrates at severe conditions, and the surfaces did not exhibit long-term stability in the acid/alkali environment, thus greatly limiting their applications in practical engineering fields. On the other hand, a very simple one-step method involving solvent evaporation to fabricate a polymer superhydrophobic surface with disordered microstructure has been reported [15-17]; however, it is easily scraped off due to the weak cohesion between the coating and substrate and the low resistance to high and low temperature alternation, in addition no long-term stability over a wide pH range (such as acid rain) was achieved. In our previous work, we firstly demonstrated that bionic superhydrophobic poly-(tetrafluoroethylene)/poly(phenylene sulfide) (PTFE/PPS) coating surfaces with long-term stability, high cohesive strength, and anti-temperature change can be prepared by a simple, inexpensive, and conventional coating-curing process [18-20]. However, the nanometer-scale structure on these superhydrophobic PTFE/PPS coating was basically cross-linking and disorderly, leading to great obstacles for further exploration on its anti-icing mechanism. Recently, Wang and coworkers have reported that robust self-cleaning coatings with well-ordered arrays were specially prepared by grafting cross-linked polymers on the silicon wafer surfaces to investigate their anti-icing mechanisms [21,22]. According to the above researches, up to now, the mechanism for self-cleaning surfaces with well-ordered polymer nano-fibers on various large-scale substrates has not been completely understood, and systematic study on it will significantly help explore new methods for polymer superhydrophobic surfaces in practical severe engineering fields.

Through the past 5 years' research, it is firstly found that bionic self-cleaning surfaces with well-ordered polymer nano-wires/fibers can be fabricated by disturbing polymer crystallization during one-step coating-curing process. Both the external macroscopic force and internal microscopic force interferences on polymer aggregates can significantly affect the nucleation and crystal growth of polymer chains under various cooling conditions. Orderly polymer nano-fibers (5 to 10 μm in length/100 nm in width) with a certain direction are obtained due to an external macroscopic force ‘*F*_blow_,’ which is on the same direction as the H_2_ gas flow. This orderly MNBS structure results in the coating with superior superhydrophobicity (WCA of 170° and WSA 0° to 1°), very similar with ‘lotus effect.’ More particularly, well-ordered nano-wires and nano-bridges (1 to 8 μm in length/10 to 80 nm in width) are generated at the non-continuous zone due to an internal microscopic tensile force (*F*_T_) by severe uneven shrinkage of adjacent continuous phases in the non-uniform medium (quenched in dry ice). The novel method for well-ordered polymer nano-fiber will provide a theoretical basis for other polymer self-cleaning surfaces with MNBS texture on various metal substrates and largely promote their practical application in many fields such as drag-reduction and anti-icing.

## Methods

### Materials and coating preparation

Bionic lotus polymer surfaces were fabricated through engineering materials, such as stainless steel or other metal substrates (Al/Cu), by using a certain volume of water-soluble PTFE emulsion and polyphenylene sulfide dispersion in mixed solvent (distilled water/ethanol/isobutyl alcohol in a volume fraction of 2:5:1), non-ionic surfactant (octylphenol polyoxyethylene ether: (C_8_H_17_-Ph-O(C_2_H_4_O)_n_H, *n* ~ 10), and industrial raw material ammonium carbonate ((NH4)_2_CO_3_) [18,20]. The steel/alumina/copper block was polished with 500^#^ and 900^#^ sand papers in turn, and then cleaned with acetone in an ultrasonic bath for 5 min. The wet coatings on stainless steel or various metal substrate blocks were prepared by spraying the coating precursors with 0.2 MPa nitrogen gas and curing at temperature 150°C for 1 h and 390°C for 1.5 h.

### External macroscopic force interference

In order to investigate the impact of external macroscopic force interference on polymer nano-fibers, pure PTFE coating (P1 coating) sample was naturally cooled to 20°C in the sintering furnace after curing at 390°C for 1.5 h. In contrast to P1 coating, H_2_ gas flow was passed into the sintering furnace during the same curing and cooling process as P1 coating for PTFE/PPS superhydrophobic coating (P2 coating) sample.

### Internal microscopic force interference

Internal microscopic force interference was introduced to further investigate controllable polymer nano-papules or nano-wires. After curing at 390°C for 1.5 h in the sintering furnace, the PTFE/PPS superhydrophobic coating samples were cooled at four different conditions, respectively, as shown in Table 1. There are three coating samples cooled in the uniform cooling mediums: the Q1 and Q2 coating were quenched in the air at room temperature (20°C) and the cryogenic liquid medium (ethanol + dry ice) at -60°C, respectively. In addition, the Q3 coating was quenched in the non-uniform cooling medium (pure dry ice cooling environment at -78.5°C).

**Table 1:** Various cooling conditions for superhydrophobic polymer coatings after curing

**Samples**	**Crystallization interference methods**	**Thermal conductivity of the mediums**[23]
Q1 coating	Quenched in the air at 20°C	*K* ≈ 0.026 [*W*/(*m K*)]
Q2 coating	Quenched in the mixture of dry ice and ethanol at -60°C	*K* ≈ 0.24 [*W*/(*m K*)]
Q3 coating	Quenched in the pure dry ice at -78.5°C	*K* ≈ 0.099 [*W*/(*m K*)]

### Characterization

Microstructures of the bionic lotus polymer coating surfaces were observed by a scanning electron microscopy (JSM-5600LV and field emission scanning electron microscopy (FE-SEM), JEOL, Akishima, Japan). Compositions of the surface of pure PTFE and PTFE/PPS coatings were analyzed by an X-ray photoelectron spectroscopy (XPS) on a VG Escalab 210 (VG Scientific, East Grinstead, UK) spectrometer with a Mg Ka X-ray source (1253.6 eV). The water static contact angle (WCA) and water sliding angle (WSA) of distilled water droplets of 5 μL on the superhydrophobic coating samples were tested by a contact angle apparatus (DSA-100, KRÜSS GmbH, Hamburg, Germany). Morphologies of the water droplets of 5 μL on the coatings were recorded with a digital camera.

## Results and discussion

### Well-ordered polymer nano-fibers by external macroscopic force interference

In our previous work, we have demonstrated a simple and conventional coating-curing process to create PTFE/PPS superhydrophobic coatings with both MNBS roughness and the lowest surface energy hydrophobic groups (-CF3) on engineering materials such as stainless steel and other metals [18,20]. However, the willow-leaf-like nanofibers are mostly cross-linking and disorderly, and the formation of these nanofibers is proposed to occur by means of a liquid-crystal ‘templating’ mechanism [24-26]. The method and mechanism for controllable fabrication of well-ordered nanofibers on the PTFE/PPS superhydrophobic coatings have always been a mystery and huge challenge for their engineering applications. In this work, we firstly found that external macroscopic force interference will help in the formation of well-ordered nanofibers.

**Figure 1:**
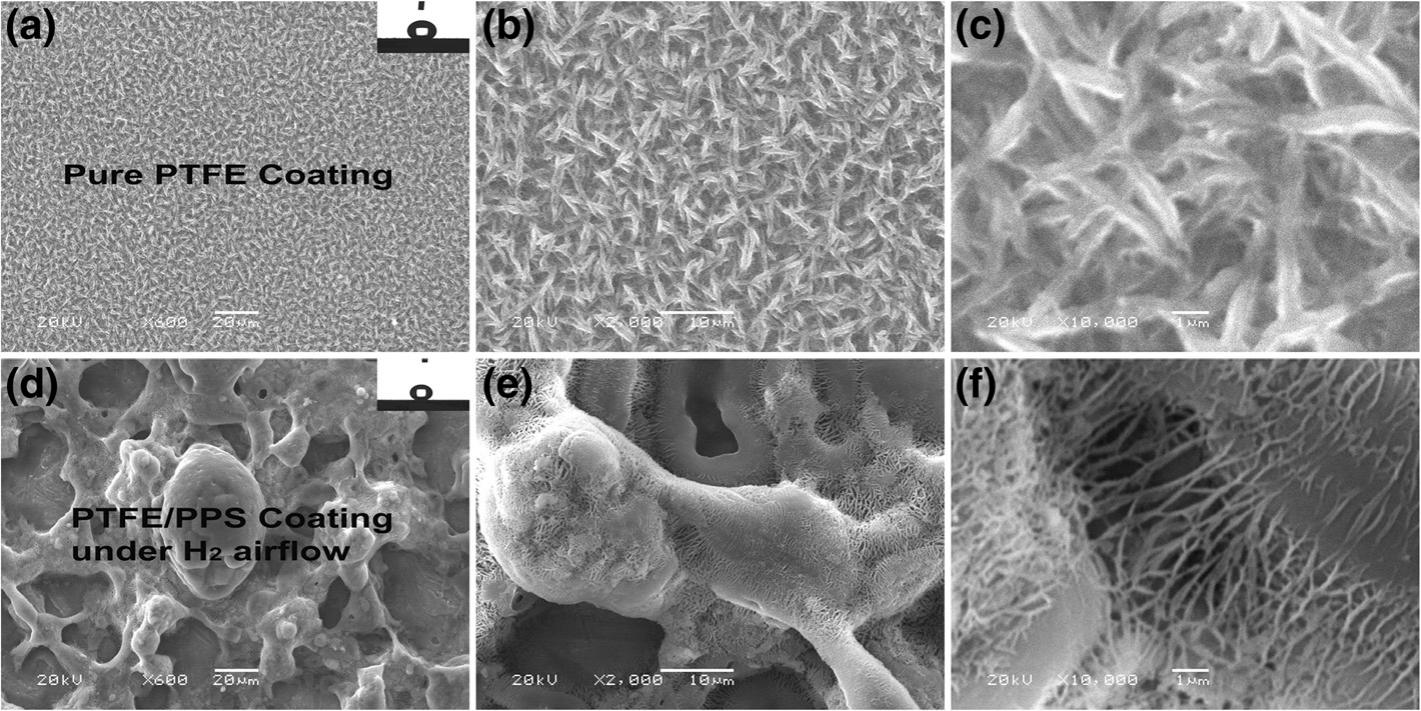
**SEM micrographs of surface microstructures of the pure PTFE and PTFE/PPS coatings.** SEM micrographs of surface microstructures with different magnifications of the pure PTFE coating surface (P1 coating) (**a** ×600, **b** ×2,000, **c** ×10,000) and PTFE/PPS superhydrophobic coating cured at 390°C under H_2_ atmosphere (P2 coating) (**d** ×600×, **e** ×2,000, **f** ×10,000). The insets show the behavior of water droplets on their surface: **(a)** WCA = 136° and **(d)** WCA = 170°.

**Figure 2:**
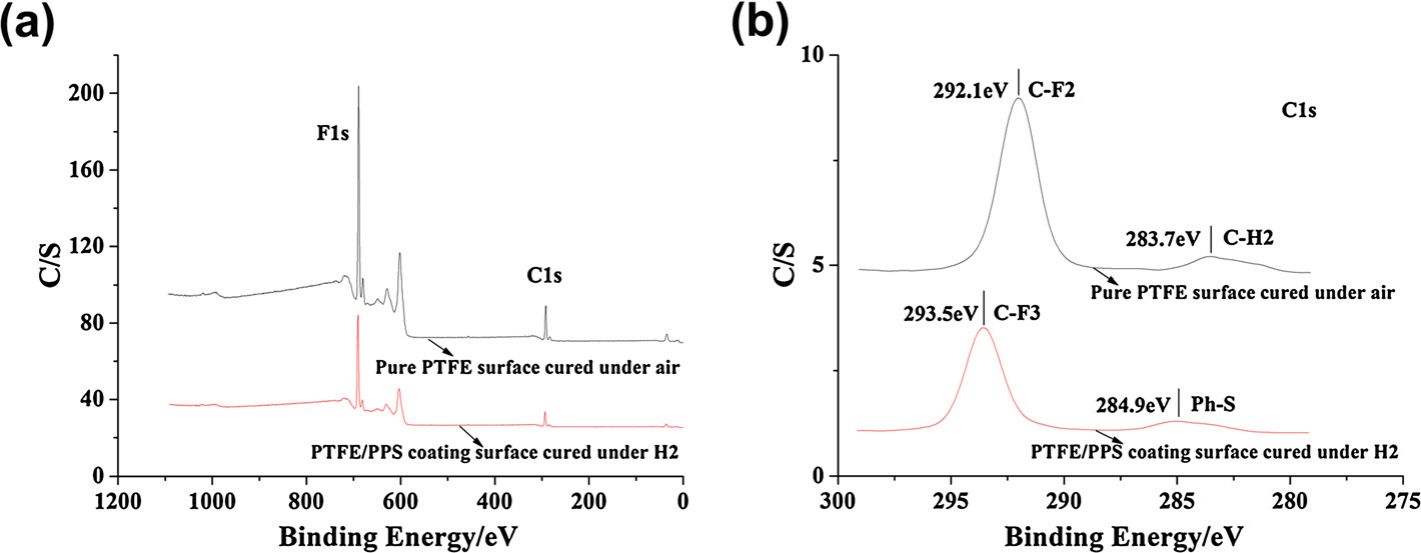
**XPS spectra for the pure PTFE and PTFE/PPS coatings.** XPS survey spectra **(a)** and XPS C1s core-level spectra **(b)** of the surfaces of pure PTFE coating (P1 coating) and PTFE/PPS superhydrophobic coating (P2 coating).

Figure 1 shows morphologies of both the pure PTFE coating and the PTFE/PPS superhydrophobic coating. Pure PTFE is prepared by curing at 390°C for 1.5 h and then naturally cooling to 20°C in the air (P1 coating). The PTFE/PPS coating is fabricated by the above process under protective atmosphere of hydrogen gas (P2 coating). Only a disordered micrometer-nanometer-scale grass and leaf-like structures (500 nm in width) were fabricated. Micropores and nano-pores formed by cross-linking of the PTFE fibers, which can be observed on the P1 coating surface (Figure 1a,b,c). The composition of the micro/nano-grass on P1 coating surface can be validated by XPS spectra (Figure 2), as shown by the strong C1s peak at 292.1 eV binding energy (C-F2) (Figure 2b) [27,28]. Based on the above nano-scale structure with only PTFE nano-fibers, P1 coating surface exhibits hydrophobicity with a WCA of 136°.

When PPS resin was added to PTFE coating (P2 coating), micrometer scale structure of porous gel network with micropapillae and isolated islands were generated. Micropores (approximately 60 μm in diameter) and micropapillae (20 to 30 μm in diameter) were scattered on the surface of porous gel network, which were similar with cauliflower pattern (Figure 1d). This porous structure could be attributed to phase separation of PPS phase [18,20,24]. Furthermore, thin and long PTFE nano-fibers with dimensions of 5 to 10 μm in length and 100 nm in width exhibited a needle-like morphology. They were distributed layer by layer on the surface of P2 coating (Figure 1e,f). The fluorine (F) was enriched at the top surface of P1 and P2 coating, as shown by the peak at 691.1 eV in the XPS survey spectra (Figure 2a). In addition, the C1s peak for P2 coating observed at 293.5 eV binding energy (C-F3) is similar to the peak at 292.1 eV (C-F2) for P1 coating (Figure 2b) [27,28]. The above data indicates the composition of the nano-fibers on P2 coating surface is mainly PTFE.

In our previous work, disorderly willow-like PTFE nano-fibers (20 to 30 μm in width) formed on the PTFE/PPS coating during the cooling process in the furnace that was exposed to air [18,20]. In our current work, these PTFE nano-fibers of P2 coating distinctly extended at a certain direction under continuous H_2_ gas flow; therefore, nano-wires and ‘nano-bridges’ formed with good directional consistency as well as uniform nano-pores (approximately 100 to 500 nm in width). In conclusion, the P2 coating surface shows superior superhydrophobicity as verified by WCA (170°) and WSA (0° to 1°) values.

Compared with P1 coating with only nano-scale fiber structure, nano-wires and nano-bridges with good directional consistency covered the microscale papillae and the interface between them on P2 coating surface, leading to formation of uniform nano-scale pores (100 to 500 nm in width). As large amount of air was captured by the nano-scale pores, the actual contact area between the water droplet and the coating surface greatly decreased [29,30]; therefore, the WCA of P2 coating increased. Moreover, the adhesion of water droplets on the orderly thin and long nano-fibers was weakened resulting in the decrease of contact angle hysteresis [29]; therefore, water droplets on P2 coating rapidly rolled down. Furthermore, the P2 coating shows better superhydrophobicity than the PTFE/PPS coating (WCA of 165° and WSA of 5°) by the same composition and curing process [20]. It is mainly because external macroscopic force interference (H_2_ gas flow) can help to form MNBS structure with well-ordered nano-bridges and uniform nano-pores (approximately 100 to 500 nm in width) (Figure 1f). Therefore, external macroscopic force interference by H_2_ gas flow during the curing and cooling processes can be a good new method for controllable fabrication of well-ordered polymer MNBS structure with lotus effect.

**Figure 3:**
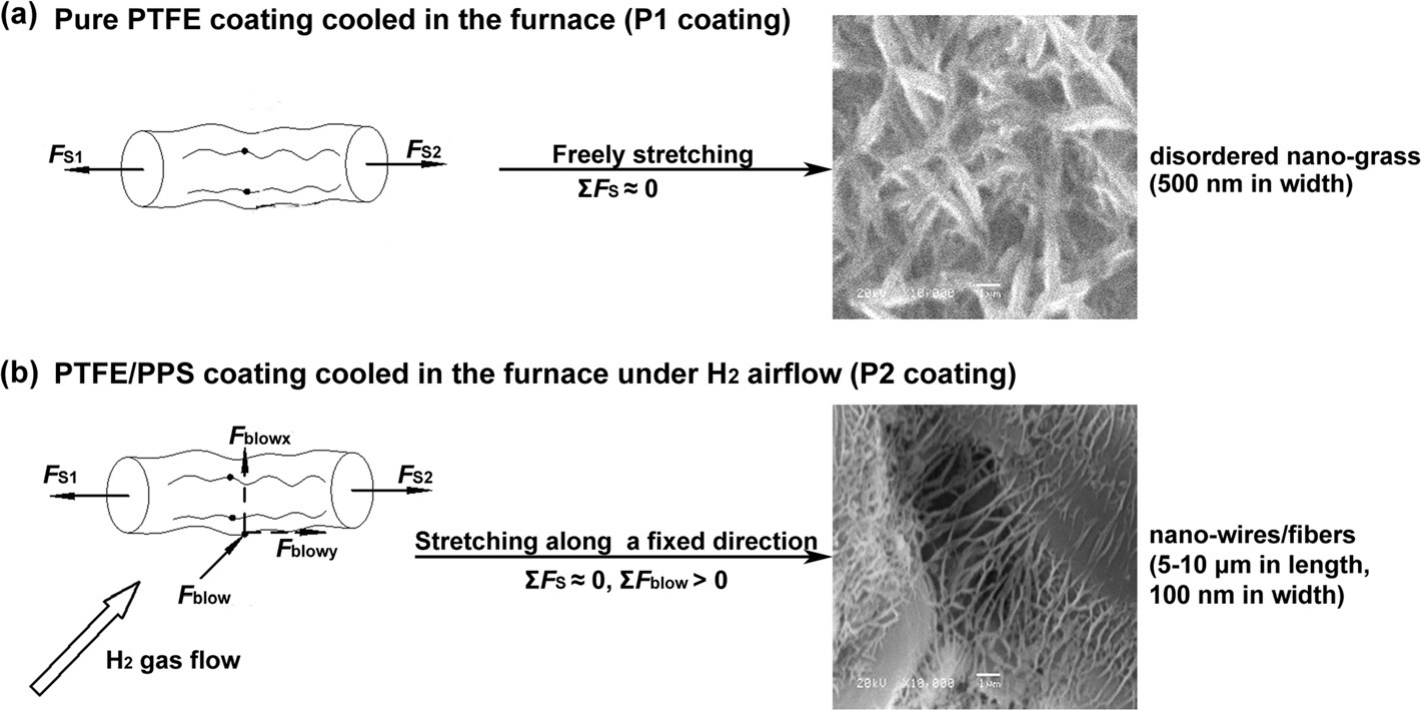
**The mechanism for well-ordered polymer nano-fibers by external macroscopic force.** The sketch map of macroscopic and microscopic forces on polymer chains during natural crystallization under protection of different atmospheres **(a, b)**: *F*_S_, a stretching force generated from natural crystallization of macromolecular chains; *F*_blow_, a microscopic force macromolecular chains derived from macroscopic H_2_ gas flow.

The speculated mechanism of P1 coating and P2 coating is shown in Figure 3. In the case of P1 coating, the temperature in the furnace was naturally cooled down from 390°C to 20°C over a period of 10 h. During the cooling process, the PTFE macromolecular chains experience nucleation and crystallization. The polymer chains stretched around and entangled with each other during crystallization process (Figure 3a), resulting in a stretching force (*F*_S_) on each PTFE macromolecular chain [31]. However, *F*_S1_ was approximately equal to *F*_S2_ as the direction of forces is opposite to each other with the similar magnitude (Figure 3a). Therefore, the stretching force (*F*_S_) could be neglected (Σ*F*s ≈ 0). Thus, PTFE macromolecular chains could stretch in an unstrained environment during the crystallization to form disordered nano-grass and nano-leaf. Compared with P1 coating, P2 coating was under protection of continuous H_2_ gas flow during the curing and cooling processes. P1 coating and P2 coating undergo the same curing and cooling process; however, a force (*F*_blow_) due to continuous H_2_ gas flow was applied on the PTFE macromolecular chains of P2 coating in addition to the stretching force *F*s (Figure 3b). The force (*F*_blow_) is function of *F*_blowx_ (perpendicular to *F*_S_) and *F*_blowy_ (parallel to *F*s), as shown in Equation 1.

(1) $$F_{\mathrm{blow}}=F_{\mathrm{blowx}}+F_{\mathrm{blowy}}$$

Thus, a new stretching force *F*_blowy_ was added to the polymer chains. Therefore, polymer nano-fibers were stretched at a greater extent compared with P1 coating along the direction of *F*_blowy,_ leading to much thinner and longer ‘nano-needles’ and nano-bridges (100 nm in width/5 to 10 μm in length).

### Polymer nano-papules or nano-wires by internal microscopic force interference

In our previous work, we have found that a higher curing temperature and longer cooling time resulted in longer crystallizing process during coating cooling process, which is beneficial to create the willow-leaf-like or wheat-haulm-leaf-like micro/nano-fiber on the atop surface of PTFE/PPS superhydrophobic coatings [20]. Moreover, the PTFE/PPS coating was hardened in H_2_O after curing at 380°C to demonstrate the mechanism of the creation of micro-nano-scale binary structures (i.e., liquid-crystal ‘templating’ mechanism). The atop surface of the PTFE/PPS coating by hardening in H_2_O was covered with micro/nano-fluorocarbon papillae textures of 200 to 800 nm in diameter compared with that produced by natural cooling in air [18,20]. However, the effect of internal microscopic force during the quenching process (crystallization process) on the nano-scale structure of the PTFE/PPS coating has still not been understood and systematically investigated.

**Figure 4:**
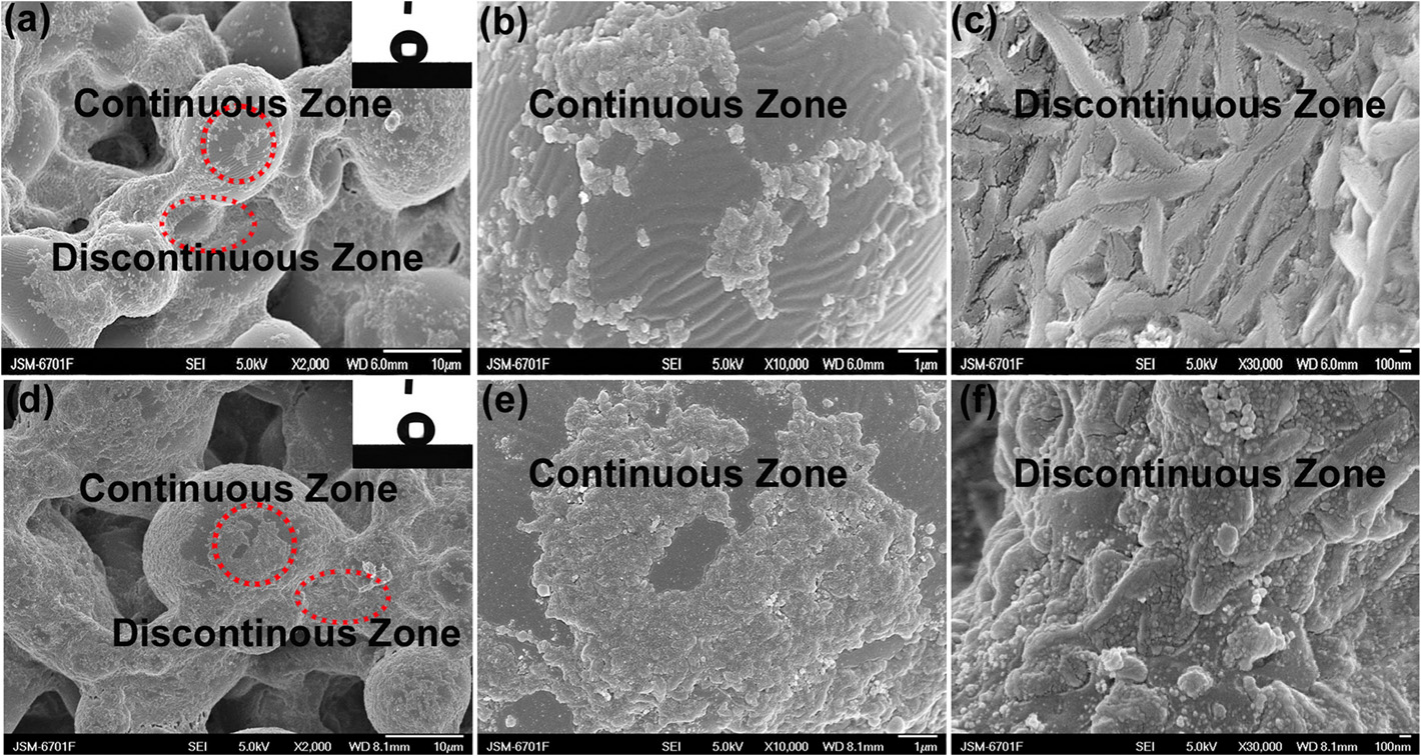
**FE-SEM micrographs for PTFE/PPS coatings via uniform cooling processes.** FE-SEM micrographs with different magnifications of surface microstructures of PTFE/PPS superhydrophobic coating cured at 390°C for 1.5 h and then quenched in air-atmosphere cooling conditions (Q1 coating) (**a** ×2,000, **b** ×10,000, **c** ×30,000) and in -60°C low temperature uniform cooling medium (Q2 coating) (**d** ×2,000, **e** ×10,000, **f** ×30,000). The continuous zone of the coatings is marked with red circles while the discontinuous zone is marked with red ellipse. The insets show the behavior of water droplets on their surfaces: (**a)** WCA = 158° and (**d)** WCA = 153°.

**Figure 5:**
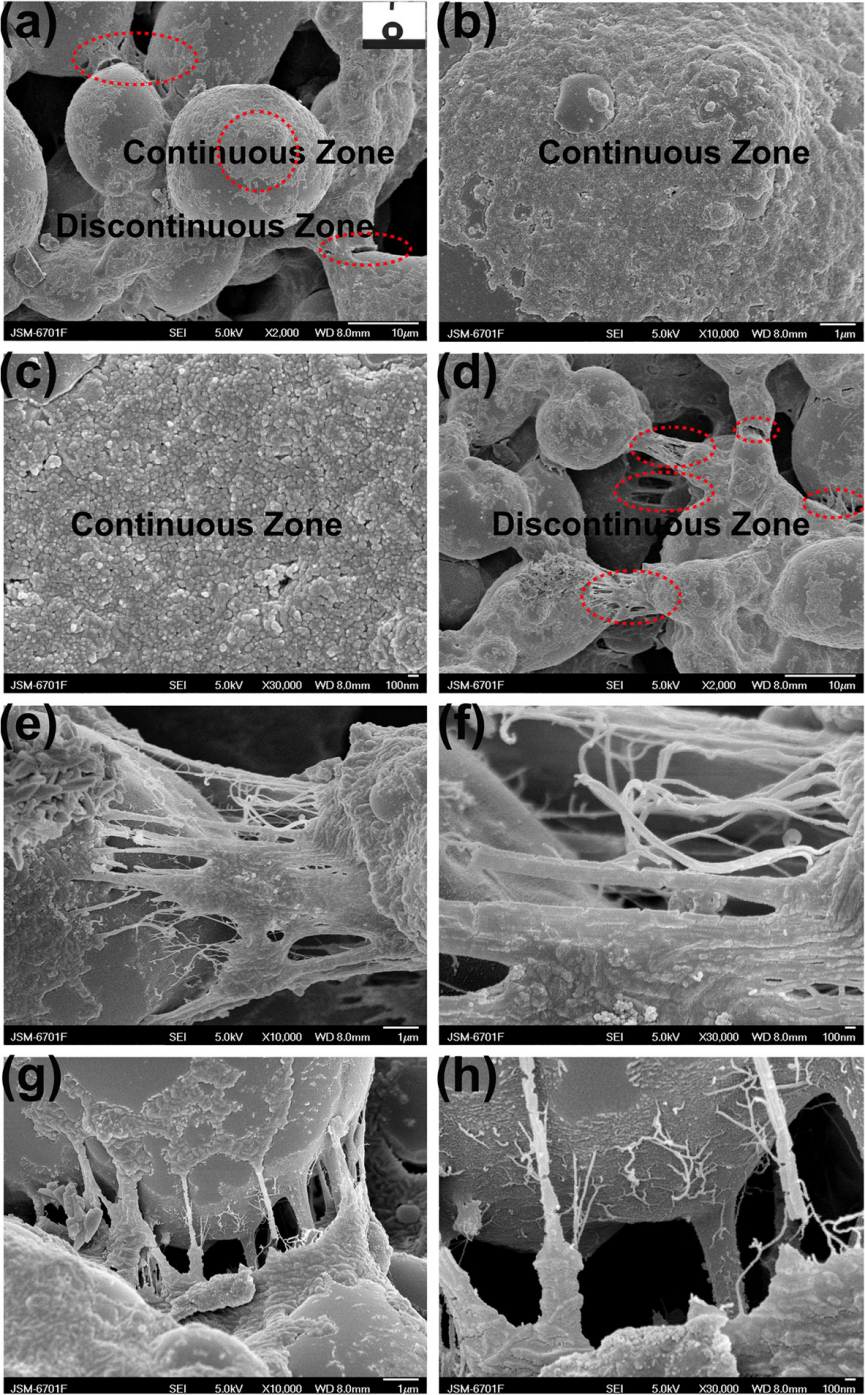
**FE-SEM micrographs for PTFE/PPS coatings via non-uniform cooling processes.** FE-SEM micrographs with different magnifications of surface microstructures of PTFE/PPS superhydrophobic coating cured at 390°C for 1.5 h and then quenched in the dry ice cooling medium (Q3 coating) (**a** ×2,000, **b** ×10,000, **c** ×30,000, **d** ×2,000, **e** ×10,000, **f** ×30,000, **g** ×10,000, **h** ×30,000).The continuous zone of the coatings is marked with red circles while the discontinuous zone is marked with red ellipse. The insets show the behavior of water droplets on Q3 coating surface: WCA = 154°.

On the basis of ‘well-ordered polymer nano-fibers by external macroscopic force (*F*_blow_) interference’ as mentioned above, the method and mechanism for orderly nano-fibers/spheres by internal microscopic force interference during the crystallization process in different cooling mediums (cooling rate) have been further systematically investigated in this work.Figure 4 shows the surface morphology of the PTFE/PPS superhydrophobic coatings fabricated by quenching in different uniform cooling mediums after curing at 390°C for 1.5 h: Q1 coating was quenched in the air at 20°C, while Q2 coating was quenched in the mixture of ethanol and dry ice at -60°C. The surface of Q1 coating also exhibits porous gel network and micropapillae structure similar with P2 coating. In addition, relatively smaller PTFE nano-spheres and papules (80 to 200 nm in diameter) were distributed uniformly and consistently on the smooth continuous surface of the micropapillae and isolated islands, as shown by the continuous zone in Figure 4b. The tangled nano-willow and nano-fiber segments were scattered on the interface surface (discontinuous zone) of the gel network and micropapillae phase (Figure 4c). Both nano-willow and nano-fiber segments are approximately 1 μm in length and 100 to 500 nm in width (Figure 4c). Q2 coating exhibits similar microstructure with Q1 coating, which is shown in Figure 4. Moreover, more uniform, dense nano-spheres and papules (approximately 60 to 150 nm in diameter) were distributed on the continuous surface of micropapillae with a relatively higher degree of overlap in comparison to Q1 coating (Figure 4d,e). Besides, shorter and wider nano-fiber segments with 100 to 500 nm in length and 200 to 400 nm in width were distributed on the rough discontinuous surface (Figure 4d,f). In addition, such MNBS texture leads to superhydrophobicity for Q1 and Q2 coating with a WCA of 158° and 153°, respectively.Furthermore, Q3 coating was hardened in the non-uniform cooling medium (pure dry ice media) at -78.5°C after curing at 390°C for 1.5 h. It can be seen that the surface of Q3 coating exhibits similar porous gel network and micropapillae structure (Figure 5a) with P2, Q1, and Q2. In addition, the PTFE nano-spheres, with 20 ~ 100 nm in diameter, were distributed most uniformly, consistently, and densely on the smooth continuous surface (continuous zone) of the micropapillae (Figure 5a,b,c). However, obvious cracks and gaps appeared on the discontinuous interface (discontinuous zone) of the gel network and micropapillae (Figure 5a,d). New polymer nano-wires were generated at the cracks or gaps between the micropapillae (Figure 5e,f,g,h). The length and width of the polymer nano-wires range from 1 to 8 μm and 10 to 80 nm, respectively. Moreover, the long PTFE nano-wires were tightly bonded on respective walls in gap forming nano-bridges (Figure 5e,f,g,h). Q3 coating has a MNBS texture with a WCA value of 154°; therefore, Q3 coating is also superhydrophobic as P2, Q1, and Q2 coating.

As the nano-scale pores between dense nano-papules and nano-spheres stacked on the micro-scale papillae of Q1, Q2 and Q3 coating were much smaller than the pores between orderly thin and long nano-fibers on P2 coating, leading to reduction of the amount of air captured by the pores; thus, the contact area between the water droplet and the coating surfaces increased [29,30], and as a result, the WCA of Q1, Q2, and Q3 coating was smaller than P2 coating by more than 10°. In addition, the adhesion of water droplets on Q1, Q2, and Q3 coating was greater than that of P2 coating, due to poor directional consistency of nano-papules on Q1, Q2, and Q3 coating. Thus, the contact angle hysteresis of water droplets increased [29], and water droplets can be placed upside down on Q1, Q2, and Q3 coating. In conclusion, polymer surfaces with nano-fiber MNBS texture generated by external macroscopic force interference possessed superior non-wettability and superhydrophobicity compared with polymer surfaces with ‘nano-papules MNBS texture’ obtained by internal microscopic force interference.

**Figure 6:**
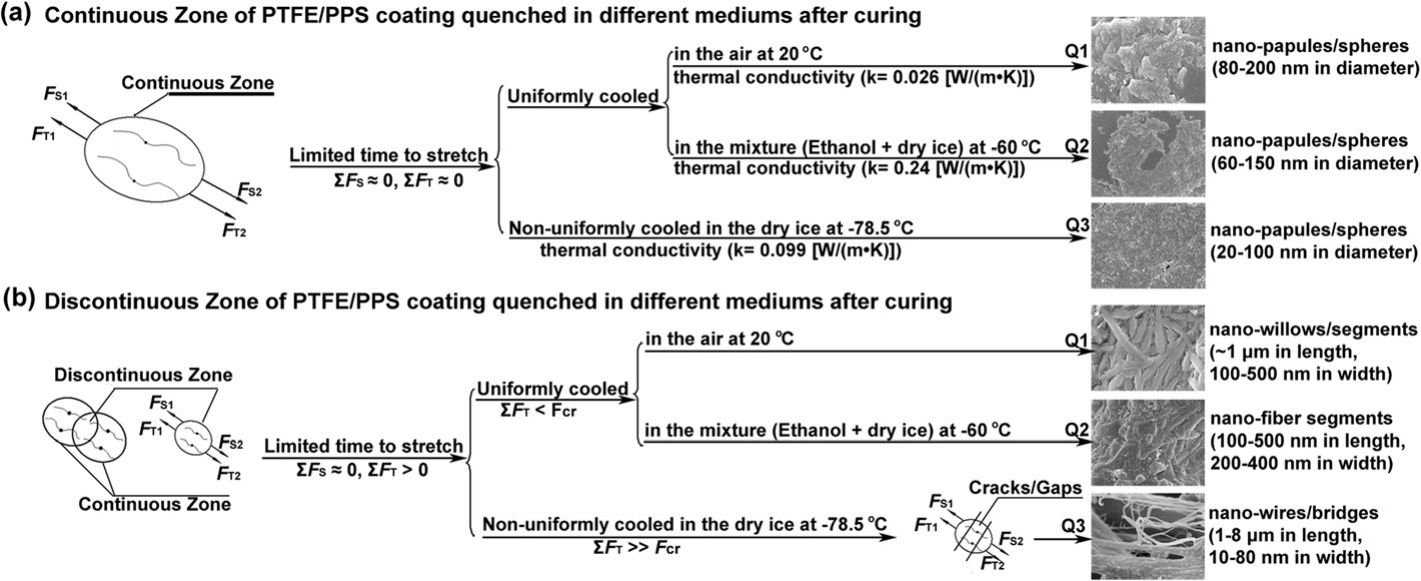
**The mechanism for polymer nano-papules or nano-wires by internal microscopic force.** The sketch map for mechanism of nano-papules, nano-segments, and nano-wires structures by internal microscopic force interferences (*F*_S_ and *F*_T_) under uniform and non-uniform cooling conditions **(a, b)**: *F*_S_, a stretching force generated from natural crystallization of macromolecular chains; *F*_T_, a new tensile force derived from the shrinkage of surrounding macromolecular chains when the temperature dramatically decreased.

Mechanism for controllable polymer nano-spheres/papules, nano-wires/fibers fabricated by disturbing crystallization process under different cooling conditions are shown in Figure 6, and the surface composition of Q1, Q2, and Q3 coating can be seen in Additional file 1: Figure S1. When the Q1 coating was quenched in the air, the PTFE aggregates (macromolecular chains) were instantly surrounded by the air molecules at 20°C (Table 1 and Figure 4). In this condition, the crystallization process of PTFE aggregates were suppressed [32], leading to the formation of fluorocarbon nano-papules on the smooth continuous surface of micropapillae (Figure 4a,b). However, rough discontinuous interfaces (discontinuous zone) of the gel network observed on Q1 coating surface (Figure 4a,c) have higher interfacial energy and longer cooling time in comparison to the continuous zone [31,33]. It is believed that high interfacial energy helps in the nucleation process and crystal growth of the polymer aggregates [33], and therefore, both thermal motion of polymer aggregates and the degree of entanglement of PTFE aggregates in the discontinuous zone in comparison to the continuous zone were enhanced, resulting in the formation of both nano-willow and nano-fiber segments.

Compared to Q1 coating, similar crystallization process took place in Q2 coating. The temperature of Q2 coating was dramatically reduced to about -60°C within just a few seconds (Table 1). It is believed that the cooling rate of the coating samples is closely related with the thermal conductivity of the cooling mediums. The nucleation and crystal growth processes of the PTFE aggregates were inhibited at a greater extent due to higher thermal conductivity compared to Q1 coating (Table 1) [23], as the thermal motion of PTFE aggregates were greatly suppressed, and therefore, there was not enough time for the PTFE aggregates to crystallize and grow to form nano-fibers (Figure 4d,e) [31,32]. On the other hand, there were large amount of protruding defects with high energy on the rough discontinuous interface between the gel network in Q2 coating (Figure 4d,f), which promote the nucleation and crystal growth of the PTFE aggregates [33]. Thus, polymer nano-spheres/papules coexisted with smaller nano-fiber segments at the end of the cooling process.

In comparison to Q1 and Q2 coating, the Q3 coating was quenched at -78.5°C in the non-uniform medium (pure dry ice) after the same curing process. The smallest polymer nano-papules (20 to 100 nm in diameter) were scattered most uniformly and densely on the continuous zone due to the highest cooling rate (Table 1). In addition, cracks/gaps were generated at the discontinuous interface (discontinuous zone) (Figure 5a,d), which can be attributed to shrinkage tension from adjacent continuous phase (continuous zone) during the abrupt intense cooling process. Thus, PTFE macromolecular chains covered on the discontinuous zone crystallized similar with Q1 and Q2 coating, and they were rapidly ‘spinned/stretched’ to form more slender polymer nano-wires and nano-bridges (10 to 80 nm in diameter), as shown in Figure 5e,f,g,h.

Furthermore, the impact of internal microscopic force generated in the abrupt intense cooling processes on the MNBS texture of the PTFE/PPS superhydrophobic coatings was investigated systematically. A stretching force (*F*s) was generated in the natural crystallization process for the continuous zone in Q1, Q2, and Q3 coating [31]. In addition, another tensile force (*F*_T_) was applied on the respective macromolecular chains in the continuous zone in Q1, Q2, and Q3 coating under quenching interference, as shown in Equation 2.

(2) $$F_{T}=E\times a_{l}\times \;\left(T_{0}\;-\;T_{1}\right)$$

Where *E* is Young's modulus, *a*_l_ is coefficient of linear expansion, and *T*_0_ and *T*_1_ are the initial and final temperatures, respectively [34]. The force *F*_T_ was derived from the intense shrinkage of surrounding macromolecular chains on the cooling process. As the temperature decreased at the same rate for the continuous zones during the whole quenching (crystallization) processes, *F*s and *F*_T_ were at the equilibrium state, respectively (Σ*F*s ≈ 0, Σ*F*_T_ ≈ 0); therefore, the crystallization of polymer chains at continuous zone of Q1, Q2, and Q3 coating was in an unconstrained environment similar with P1 coating. However, the crystal growth of polymer chains was different because crystallization time of Q1, Q2, and Q3 coating was much shorter than P1 coating (Table 1). Therefore, only nano-spheres/papules formed in the continuous zone for Q1, Q2, and Q3 coating. Moreover, increasing the cooling rate gradually from Q1 to Q3 coating (Table 1) resulted in a size reduction of polymer nano-spheres with a higher degree of overlap.

On the other hand, for the discontinuous zone of Q1, Q2, and Q3 coating (Figures 4 and 5) between the porous gel network and micropapillae, the nucleation and crystal growth of polymer chains were promoted because of high interfacial energy [33]. At the same time, the cooling time in the discontinuous zone was longer than the continuous zone because of less exposure in the cooling medium. Although a tensile force (*F*_T_) was generated by the uneven shrinkage from adjacent continuous phase of the coatings under the quenching interference [35-37], *F*_T_ was much smaller than the critical value (*F*_cr_) for both Q1 and Q2 coating. Thus, the crystallization process of polymer chains was dominated by the crystallization driving force and crystallization time [32,38]; therefore, nano-willow and nano-fiber segments were obtained in the discontinuous zone of Q1 coating, while nano-spheres/papules coexisted with smaller nano-fiber segments in the discontinuous zone of Q2 coating.

However, when Q3 coating was quenched in a non-uniform medium interference, the polymer chains at discontinuous zone suffered much larger tensile force *F*_T_ than the discontinuous zone of Q1 and Q2 coating, due to the significant temperature difference between the continuous zone and discontinuous zone (Table 1). The tensile force *F*_T_ was large enough (*F*_T_> > *F*_cr_, and Σ*F*_T_> > 0) to pull the discontinuous zone off to form cracks and gaps, as shown by the discontinuous zone in Figures 5e,f,g,h and 6b. Therefore, nano-wires and nano-bridges can be formed by spinning polymer aggregates (Figure 5e,f,g,h).

As mentioned above, both macroscopic force interference and internal microscopic force interference will significantly affect the crystallization of polymer chains under different conditions. The MNBS texture and surface behaviors of these coatings are summed in Table 2. In comparison to disordered nano-grass structure of P1 coating, PTFE nano-fibers (5 to 10 μm in length/100 nm in width) with good directional consistency covered the microscale papillae (continuous zone) and the interface (discontinuous zone) between them on P2 coating surface, due to external macroscopic force interference by H_2_ gas flow (Figure 3b). Since large amount of air was captured by the nano-scale pores and the adhesion of water droplets on the orderly thin and long nano-fibers was significantly weakened [29,30], the P2 coating surface shows superior superhydrophobicity (a WCA of 170° and a WSA of 0° to 1°). On the other hand, as the internal microscopic force interference (cooling rate) gradually increased, smaller and smaller PTFE nano-spheres and papules (80 to 200 nm, 60 to 150 nm, and 20 to 100 nm in diameter) were distributed uniformly and consistently on the smooth continuous surface (continuous zone) of Q1 coating (quenched in the air at 20°C), Q2 coating (quenched in the mixture of ethanol and dry ice at -60°C), and Q3 coating (quenched in pure dry ice at -78.5°C), respectively (Figures 4b,e and 5c). In addition, much shorter and wider nano-scale segments were distributed on the rough discontinuous surface (discontinuous zone) of Q1 and Q2 coating compared with P1 coating. Moreover, PTFE macromolecular chains were rapidly ‘spinned/stretched’ to new nano-scale ‘bridges’ (1 to 8 μm in length/10 to 80 nm in width) by a great microscopic tensile force at discontinuous interface (discontinuous zone) of Q3 coating (Figure 5e,f,g,h). As much smaller nano-papules/spheres with poor directional consistency stacked densely on the continuous zone of Q1, Q2, and Q3 coating, the contact area between the water droplet and the coating surfaces increased at some extent, and the adhesion of water droplets on Q1, Q2, and Q3 coating was greater than that of P2 coating [29,30]. As a result, the WCA of Q1, Q2, and Q3 coating was smaller than P2 coating by more than 10°, and water droplets can be placed upside down on these coatings. In summary, polymer surfaces with nano-fiber MNBS texture by external macroscopic force interference possessed superior superhydrophobicity compared to ‘nano-papules MNBS texture’ by internal microscopic force interference, and introducing external macroscopic force interference by H_2_ gas flow to the curing and cooling processes could be a good method for controllable fabrication of well-ordered polymer nano-fiber MNBS texture with lotus effect.

**Table 2:** MNBS texture and surface behaviors of the coatings

**Samples**	**MNBS texture**		**WCAs (degrees)**	**WSAs (degrees)**
	**Continuous zone**	**Discontinuous zone**		
P1 coating	Disordered nano-grass (500 nm in width)	-	136	-
P2 coating	Well-ordered nano-fibers (5 to 10 μm in length/100 nm in width)	Well-ordered nano-fibers (5 to 10 μm in length/100 nm in width)	170	0 to 1
Q1 coating	Nano-scale spheres/papules (80 to 200 nm in diameter)	Willow-like nano-scale segments (approximately 1 μm in length/100 to 500 nm in width)	158	-
Q2 coating	Nano-scale spheres/papules (60 to 150 nm in diameter)	Nano-scale fiber segments (100 to 500 nm in length/200 to 400 nm in width)	153	-
Q3 coating	Nano-scale spheres/papules (20 to 100 nm in diameter)	Orderly nano-scale wires/bridges (1 to 8 μm in length/10 to 80 nm in width)	154	-

## Conclusions

By disturbing crystallization during one-step coating-curing process, bionic lotus surfaces with controllable polymer nano-spheres/papules, nano-wires/fibers were firstly fabricated. It is demonstrated that both macroscopic force interference and internal microscopic force interference on polymer aggregates under different cooling conditions will significantly affect the crystallization of polymer chains. Polymer chains stretched and elongated freely to form a disordered micro-nano-scale grass/leaf-like morphologies in pure PTFE coating (P1 coating), while orderly polymer nano-fibers (100 nm in length/5 to 10 μm in width) with a certain direction are obtained by the force *F*_blow_ along the direction of H_2_ gas flow. During the quenching process in the uniform and non-uniform mediums, nano-papules/spheres (20 to 200 nm in diameter) formed in the continuous zone, while polymer aggregates are partially stretched to nano-fiber segments (1 μm in length/100 to 500 nm in width) during the crystallization process in the discontinuous zone.

However, by polymer crystallization interference in the non-uniform medium, the polymer chains at discontinuous zone of Q3 coating suffered much greater tensile force (*F*_T_) in comparison to Q1 and Q2 coating_,_ which can be attributed to the temperature difference between the continuous zone and discontinuous zone. The tensile force was large enough (*F*_T_> > *F*_cr_ and Σ*F*_T_> > 0) to generate cracks and gaps in the discontinuous zone for Q3 coating. Therefore, nano-wires and nano-bridges (1 to 8 μm in length/10 to 80 nm in width) formed. We bring a novel perspective to controllable polymer nano-fibers; this study will provide a theoretical basis for polymer superhydrophobic surface with MNBS texture and promote development of polymer superhydrophobic surfaces in many engineering fields such as drag reduction and anti-icing.

## Abbreviations

WCA: water contact angle; WSA: water sliding angle; MNBS: micrometer-nanometer-scale binary structure; PTFE: poly-(tetrafluoroethylene); PPS: poly(phenylene sulfide); SEM: scanning electron microscopy; XPS: X-ray photoelectron spectroscopy.

## Competing interests

The authors declare that they have no competing interests.

## Authors’ contributions

ZL, ZZ, and WL gave the guidance; QY, ST, YL, and YW participated in the experiments; and QY and ZL analyzed the data and contributed to the draft of the manuscript. All authors read and approved the final manuscript.

## Supplementary Material

Additional file 1: Figure S1 XPS survey spectra (a) and XPS C1s core-level spectra (b) of the surfaces of PTFE/PPS superhydrophobic coating samples cured at 390°C for 1.5 hours and then quenched in: air-atmosphere (2°C) cooling conditions (Q1 coating), low temperature (-60°C) uniform cooling medium (Q2 coating), and low temperature pure dry ice (20°C) non-uniform cooling medium (Q3 coating). Click here for file
