# Supplementary material for: Well-ordered polymer nano-fibers with self-cleaning property by disturbing crystallization process
Source: Nanoscale Res Lett. 2014 Jul 15;9(1):352. doi: 10.1186/1556-276X-9-352 (PMC4106910; doi:10.1186/1556-276X-9-352)
Supplement: Additional file 1: Figure S1 — XPS survey spectra (a) and XPS C1s core-level spectra (b) of the surfaces of PTFE/PPS superhydrophobic coating samples cured at 390°C for 1.5 hours and then quenched in: air-atmosphere (2°C) cooling conditions (Q1 coating), low temperature (-60°C) uniform cooling medium (Q2 coating), and low temperature pure dry ice (20°C) non-uniform cooling medium (Q3 coating). [file 1556-276X-9-352-S1.doc]

**Support Information:**

**Figure S1**. XPS survey spectra (a) and XPS C1s core-level spectra (b) of the surfaces of PTFE/PPS superhydrophobic coating samples cured at 390°C for 1.5 hours and then quenched in: air-atmosphere (20 ^o^C) cooling conditions (Q1 coating), low temperature (-60 ^o^C) uniform cooling medium (Q2 coating), and low temperature pure dry ice (20 ^o^C) non-uniform cooling medium (Q3 coating).
